# A Comparative Analysis of the Molecular Features of MANF and CDNF

**DOI:** 10.1371/journal.pone.0146923

**Published:** 2016-01-28

**Authors:** Junpei Norisada, Yoko Hirata, Fumimasa Amaya, Kazutoshi Kiuchi, Kentaro Oh-hashi

**Affiliations:** 1 United Graduate School of Drug Discovery and Medical Information Sciences, Gifu University, 1–1 Yanagido, Gifu 501–1193, Japan; 2 Department of Chemistry and Biomolecular Science, Faculty of Engineering, Gifu University, 1–1 Yanagido, Gifu 501–1193, Japan; 3 Department of Anesthesiology, Kyoto Prefectural University of Medicine, Kawaramachi-Hirokoji, Kamikyo-ku, Kyoto 602–0841, Japan; University of Toronto, CANADA

## Abstract

Cerebral dopamine neurotrophic factor (CDNF) is a paralogous protein of mesencephalic astrocyte-derived neurotrophic factor (MANF). Both proteins have been reported to show a common cytoprotective effect on dopaminergic neurons as a secretory protein containing the KDEL-like motif of the ER retrieval signal at the C-terminus, RTDL in MANF and [Q/K]TEL in CDNF among many species, although functions of paralogous proteins tend to differ from each other. In this study, we focused on post-translational regulations of their retention in the endoplasmic reticulum (ER) and secretion and performed comparative experiments on characterization of mouse MANF and mouse CDNF according to our previous report about biosynthesis and secretion of mouse MANF using a NanoLuc system. In this study, co-expression of glucose-regulated protein 78 kDa (GRP78), KDEL receptor 1 or mutant Sar1 into HEK293 cells similarly decreased MANF and CDNF secretion with some degree of variation. Next, we investigated whether CDNF affects the secretion of mouse cysteine-rich with EGF-like domains 2 (CRELD2) because mouse wild-type (wt) MANF but not its KDEL-like motif deleted mutant (ΔC_MANF_) was found to promote the CRELD2 release from the transfected cells. Co-expressing CRELD2 with wt or ΔC CDNF, we found that CDNF and ΔC_MANF_ hardly elevated the CRELD2 secretion. We then investigated effects of the four or six C-terminal amino acids of MANF and CDNF on the CRELD2 secretion. As a result, co-transfection of mouse CDNF having the mouse MANF-type C-terminal amino acids (CDNF_RTDL_ and CDNF_SARTDL_) increased the CRELD2 secretion to a small extent, but mouse CDNF having human CDNF-type ones (CDNF_KTEL_ and CDNF_HPKTEL_) well increased the CRELD2 secretion. On the other hand, the replacement of C-terminal motifs of mouse MANF with those of mouse CDNF (MANF_QTEL_ and MANF_YPQTEL_) enhanced the CRELD2 secretion, and the mouse MANF having human CDNF-type ones (MANF_KTEL_ and MANF_HPKTEL_) dramatically potentiated the CRELD2 secretion. These results indicate that the secretion of mouse MANF and mouse CDNF is fundamentally regulated in the same manner and that the variation of four C-terminal amino acids in the MANF and CDNF among species might influence their intracellular functions. This finding could be a hint to identify physiological functions of MANF and CDNF.

## Introduction

Various types of stress are considered to be associated with the onset and progression of neurodegenerative diseases, including Parkinson’s disease and Alzheimer’s disease. Under pathophysiological conditions, cellular stresses disrupt appropriate functions of endoplasmic reticulum (ER) and cause the accumulation of misfolded and/or unfolded proteins in the ER. This predicament, termed ER stress [1, 2], activates unfolded protein responses (UPR), which are mediated by three transmembrane ER-resident (or localized) proteins, PERK [3], IRE1 [4] and ATF6 [5, 6]. UPR attenuate ER stress by suppressing translation of mRNAs, inducing chaperones and reinforcing endoplasmic reticulum associated degradation (ERAD) [7]. However, the excessive ER stress causes cell death [8]. Mesencephalic astrocyte-derived neurotrophic factor (MANF) has been reported to be a downstream target of ATF6α, ATF6β and sXBP1 [9–12] and is induced in parallel with ER-resident chaperones [9–13] and even pro-apoptotic factor such as growth arrest- and DNA damage-inducible gene 153 (GADD153) in the UPR [14].

MANF was originally identified as arginine-rich, mutated in early stage of tumors (Armet), a protein with a high mutation rate in various tumors [15, 16]. Petrova *et al*. demonstrated that MANF, a secretory protein from a rat mesencephalic type-1 astrocytic cell line, is identical with Armet and performs a selective neurotrophic effect on dopaminergic neurons [17]. In this study, Armet is referred to as MANF, even though the precise mechanisms by which it protects both neuronal cells and non-neuronal cells from cell death remain unclear [17–22]. Accordingly, it is thought that the elucidation of physiological actions of MANF is useful for establishing a therapy for neurodegenerative diseases, including Parkinson’s disease and Alzheimer’s disease.

Cerebral dopamine neurotrophic factor (CDNF) is a vertebrate-specific paralog of MANF [17]. It is reported that CDNF also showed a cytoprotective effect on 6-OHDA-induced Parkinson’s disease model rats [23], and that the expression level of CDNF mRNA was constitutive and uninfluenced by ER stress [19]. However, the transcriptional regulation of CDNF gene remains to be determined. Although paralogous proteins tend to show different functions, the only common function reported between these proteins is the cytoprotective effect on dopamine neurons. Consequently, the clarification of distinctions and similarities between MANF and CDNF is considered to give insights into their functions. However, a comparative investigation of MANF and CDNF has not been reported, except for their structural comparison in *Drosophilia* [24].

In this study, we performed the comparative consideration for secretory regulation of MANF and CDNF because we have investigated the mechanism of MANF secretion by developing a highly sensitive and quantitative assay for the measurement of MANF secretion using a small luciferase, NanoLuc (NL), in previous research [25, 26]. In addition, we have recently demonstrated that MANF-overexpression potentiates the secretion of cysteine-rich with EGF-like domains 2 (CRELD2) using several MANF mutants [27, 28]. Based on our previous studies [26–30], we compared the secretory regulation of mouse MANF and mouse CDNF by co-transfection of glucose-regulated protein 78 kDa (GRP78), KDEL receptor1 (KDEL-R1) and mutant-Sar1 and revealed their different actions on the mouse CRELD2 secretion by focusing on their four or six C-terminal amino acids.

## Materials & Methods

### 1. Construction of plasmids

For the preparation of each mouse MANF and mouse CDNF constructs, the wild-type (wt) MANF and CDNF genes were cloned from cDNA derived from a mouse neuroblastoma cell-line, Neuro2a, using RT-PCR and inserted into the pcDNA3.1 vector (Life Technologies, U.S.A.) as described previously [27, 29]. To construct the indicated tagged-MANF and -CDNF, DsRed2, EGFP, Flag-epitope and NL [25, 26] were inserted downstream of the putative signal peptide sequence (MANF, 23 amino acids and CDNF, 24 amino acids) of full length MANF or CDNF and cloned into the pcDNA3.1 vector as described previously [26, 28]. Mouse MANF and CDNF mutants that were lacking their four C-terminal amino acids or that had their four or six C-terminal amino acids exchanged were also amplified and cloned into the pcDNA3.1 vector as described previously [28]. Genes encoding mouse wtGRP78 and wtCRELD2 were obtained from DNAFORM (RIKEN, Japan) and each fragment was cloned into the pcDNA3.1 vector as previously described [30]. An HA-tagged mutant-Sar1 (H79G) construct was kindly provided by Dr. Wei Liu and Dr. Jennifer Lippincott-Schwartz [31]. A *myc*-tagged KDEL-R1 was kindly gifted by Dr. Lloyd Ruddock [32].

### 2. Cell culture and treatment

HEK293 and COS7 cells were maintained in Dulbecco’s modified Eagle’s minimum essential medium containing 8% fetal bovine serum. To detect the indicated proteins by western blot analysis and fluorescent microscopy, cells were seeded into 12-well plate. For luciferase analysis, cells were seeded into a 48-well plate, grown to semi-confluence and used for subsequent experiments. Transfection of the indicated plasmids was performed using Lipofectamine-Plus reagents (Life Technologies, U.S.A.) and PEI-MAX (Polysciences, U.S.A.) as described previously [27, 33].

### 3. Luciferase assay

Twenty-four hours after transiently overexpressing the indicated constructs, cells were incubated in the serum-free medium for 4 h at 37°C, then culture medium and cell lysate were collected and extra- and intracellular luciferase activities were calculated as described previously [26].

### 4. Western blot analysis

Cells in each well were lysed with homogenate buffer [20 mM Tris-HCl (pH 8.0) containing 137 mM NaCl, 2 mM EDTA, 10% glycerol, 1% Triton X-100, 1 mM PMSF, 10 μg/ml leupeptin and 10 μg/ml pepstatin A] as described previously [27]. After determining the protein concentrations by a Bradford Reagent (BioRad Laboratories, U.S.A.), cell lysates were dissolved in SDS-Laemmli sample buffer [62.5 mM Tris-HCl (pH 6.8), 2% SDS and 10% glycerol], and equal amounts of cell lysates in each experiment were prepared. To detect MANF, CDNF and CRELD2 proteins in the culture medium, equal amount of each culture medium was resuspended in SDS-Laemmli sample buffer. In each experiment, equal amount of each sample from lysate and culture medium were separated on 8.0–15.0% SDS-polyacrylamide electrophoresis gels, blotted onto polyvinylidene difluoride membranes (GE Healthcare Bioscience, U.S.A.) and identified by enhanced chemiluminescence using antibodies against MANF, CDNF, CRELD2, the Myc-epitope, the Flag-epitope or actin. The primary antibodies used are as follows: anti-actin antibody (Calbiochem, U.S.A.); anti-CDNF and anti-CRELD2 antibodies (R&D Systems, U.S.A.); anti-Flag antibody (M2, Sigma-Aldrich, U.S.A.); anti-GRP78 antibody (Cell Signaling, U.S.A.); and anti-MANF antibodies (abcam, U.K. and R&D Systems, U.S.A.); anti-Myc antibody (Santa Cruz Biotechnology, U.S.A). More than three independent cultures were performed to confirm reproducibility and the sample number was indicated in each of figure legends. The amounts of the secreted CRELD2 were analyzed by Image J software (National Institutes of Health. U.S.A.) and normalized by the value from mock transfected cells.

### 5. Fluorescent images

COS7 cells were seeded on poly-D-lysine coated glass coverslips and transfected with SP-EGFP-MANF, SP-DsRed2-MANF, SP-EGFP-CDNF and/or SP-DsRed2-CDNF. Forty-eight hours after transfection, cells were washed using PBS and fixed with 4% paraformaldehyde for 15 min. After washing with PBS, the cells were mounted with PermaFluor Mountant Medium (Thermo Fisher Scientific, U.S.A.) and fluorescent images were obtained by fluorescent microscopy using 470 nm and 540 nm filters (BZ-9000; KEYENCE, Japan) as described previously [28, 30].

### 6. Statistical analysis

The results are expressed as the mean ± SEM of the indicated number. The statistical analyses were carried out by One-way ANOVA following Turkey’s Multiple Comparison Test or Student’s t-test. p < 0.05 was considered to be statistically significant.

## Results

Our group and others have reported the secretory regulation of MANF; however, the intracellular transport and regulation of CDNF secretion is not fully characterized [20, 26, 29, 34–36]. Accordingly, we focused on the secretory regulation of mouse CDNF and clarified differences between CDNF and MANF secretion on the molecular level based on our previous study about MANF [26, 29].

**Fig 1:**
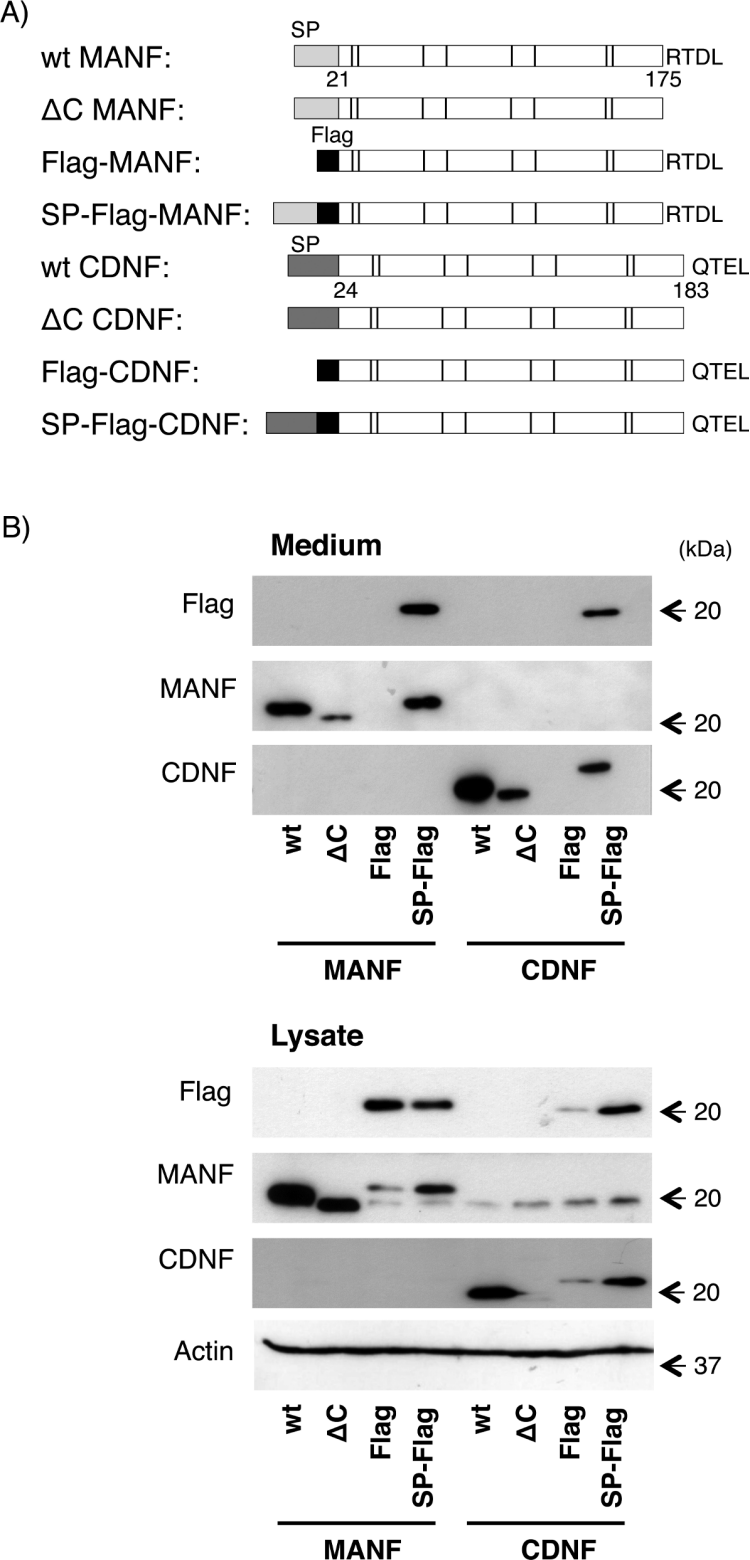
Intracellular expression and extracellular secretion of MANF and CDNF in HEK293 cells. (A) Schematic representation of the mouse MANF and CDNF expression constructs used in this study. SP indicates a signal peptide at the N-terminus of each protein. The cysteines are indicated by bars. The four C-terminal amino acids, RTDL and QTEL, putative ER localization signals at their C-termini are shown in capital letters. (B) Western blot analysis of wild-type and modified MANF and CDNF overexpressed in HEK293 cells. Twenty-four hours after transfection of each indicated construct into the cells, the culture medium was replaced with fresh serum-free DMEM, and the cells were incubated for an additional 12 h. The amounts of the indicated proteins in the cell lysate and culture medium were detected by western blot analysis using antibodies against Flag-epitope, MANF, CDNF and actin as described in the Materials and Methods. Representative data of three independent experiments were shown.

First, to compare the secretory profile of both proteins, we prepared expression vectors of mouse MANF and mouse CDNF, which have a common Flag-epitope just behind their signal peptide sequences at the N-terminus (SP-Flag-MANF and SP-Flag-CDNF, respectively, Fig 1A). As shown in Fig 1B, the secretion levels of Flag-tagged MANF and CDNF were nearly identical. Consistent with the case of MANF [29], CDNF lacking the signal peptide (Flag-CDNF) was also not secreted into the extracellular space.

**Fig 2:**
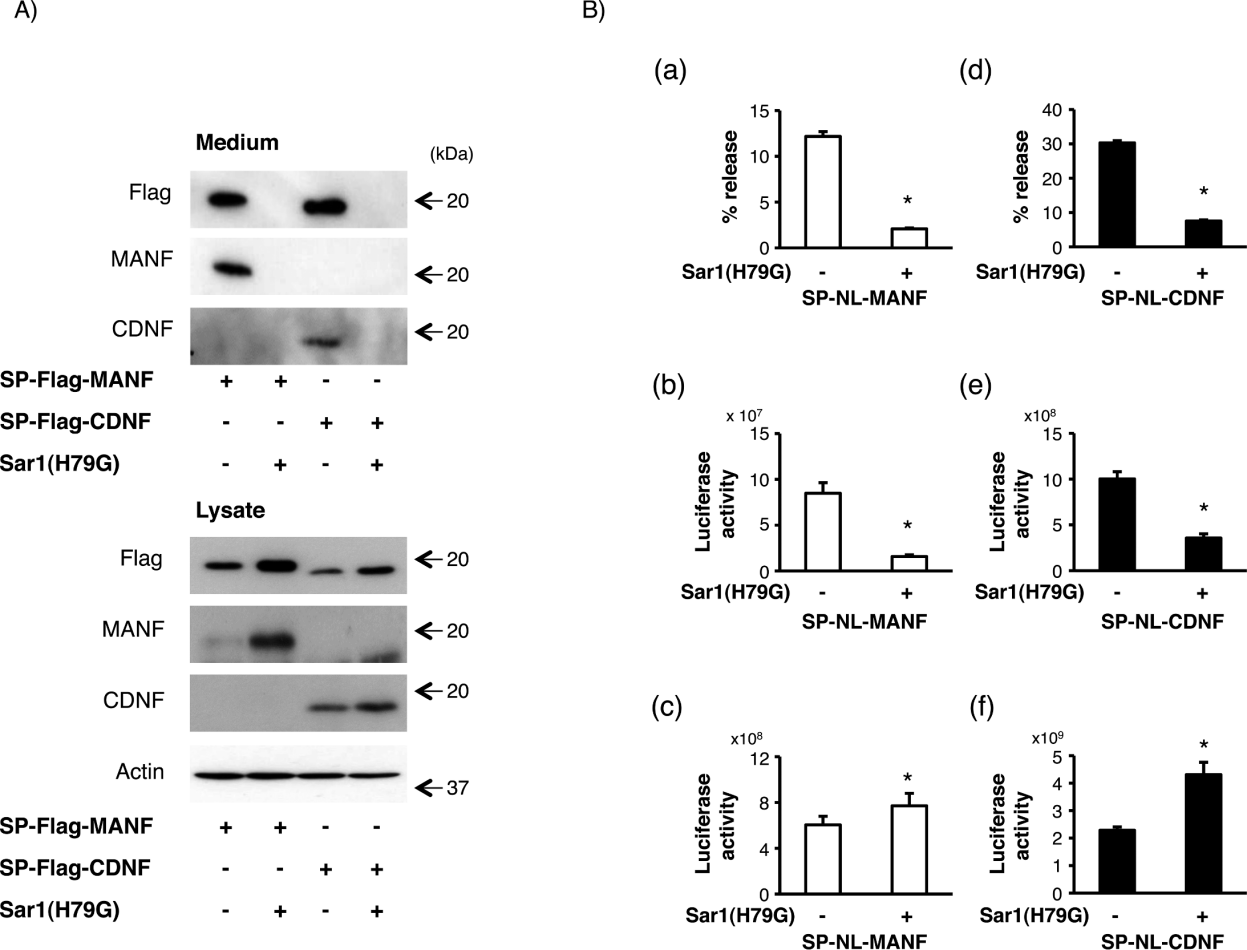
Effects of mutant-Sar1 co-expression on the secretion of MANF and CDNF from HEK293 cells. (A) Twenty-four hours after the transfection of SP-Flag-MANF or SP-Flag-CDNF with Sar1(H79G) or the empty vector (mock) into HEK293 cells, the culture medium was replaced with fresh serum-free medium and the cells were cultured for an additional 12 h. The amounts of MANF and CDNF in the cell lysate and culture medium were detected by western blot analysis as described in the Materials and Methods. Representative data of three independent cultures were shown. (B) Twenty-four hours after the transfection of SP-NL-MANF or SP-NL-CDNF with Sar1(H79G) or the empty vector (mock) into HEK293 cells, the culture medium was replaced with serum-free medium, and the cells were incubated for an additional 4 h. The culture medium (b, e) and cell lysate (c, f) from HEK293 cells expressing SP-NL-MANF or SP-NL-CDNF were collected. The luciferase activity in each sample was measured as described in the Materials and Methods. The values represent the mean ± SEM from nine independent cultures. The relative amounts of secreted SP-NL-MANF and SP-NL-CDNF in each case (a, d) were calculated from the data of their extracellular activities (b, e) and their intracellular activities (c, f), respectively. The data were analyzed by Student’s t-test to evaluate the effects of the co-expression of Sar1(H79G) on the luciferase activity. The values marked with an asterisk are significantly different from the value of the mock-transfected cells, respectively (p < 0.05).

Next, we further investigated a pathway for the intracellular transport of CDNF, as is the case of MANF. Sar1 is a critical component of COPII-coated vesicles and plays an important role in the COPII-mediated transport from the ER to the Golgi apparatus [31, 37]. We previously reported that the overexpression of mutant-Sar1, Sar1(H79G), impaired MANF secretion and that the intracellular MANF was increased in inverse proportion [29]. We therefore compared the secretion of CDNF with that of MANF by the co-transfection of Sar1(H79G). As shown in Fig 2A, the secretory profile of SP-Flag-CDNF was similar to that of SP-Flag-MANF, which showed a decrease in the extracellular level and an increase in the intracellular level. Recently, we developed a more convenient and quantitative assay for determining the biosynthesis and secretion of MANF using a highly active and small luciferase, NanoLuc [25, 26]. We then adopted this system to confirm the secretory profile of MANF and CDNF more quantitatively (Fig 2B). Consistent with the results obtained from the western blot analysis, the overexpression of Sar1(H79G) decreased both of the secretion levels (17.5 ± 1.4% and 24.9 ± 1.2%, respectively) in inverse proportion to the increase in their intracellular levels (126.0 ± 5.5% and 185.1 ± 10.5%, respectively) (Table 1).

**Table 1:** The relative amounts of SP-NL-MANF and SP-NL-CDNF from HEK293 cells co-transfected with mock or Sar1(H79G).

	% release		Medium		Lysate	
	mock	Sar1(H79G)	mock	Sar1(H79G)	mock	Sar1(H79G)
**MANF**	100.0 ± 1.4	17.5 ± 1.4	100.0 ± 3.4	20.0 ± 1.9	100.0 ± 3.5	126.0 ± 5.5
**CDNF**	100.0 ± 1.4	24.9 ± 1.2	100.0 ± 3.5	34.6 ± 2.4	100.0 ± 2.4	185.1 ± 10.5

Values show the relative luciferase activities represented in Fig 2B. The relative amounts of secreted SP-NL-MANF and SP-NL-CDNF in each case (Fig 2B a, d) were calculated from the extracellular (Fig 2B b, e) and intracellular (Fig 2B c, f) activities of SP-NL-MANF and SP-NL-CDNF, respectively. Each value represents the mean ± SEM from nine independent cultures and was expressed as a percentage of mock cells.

**Fig 3:**
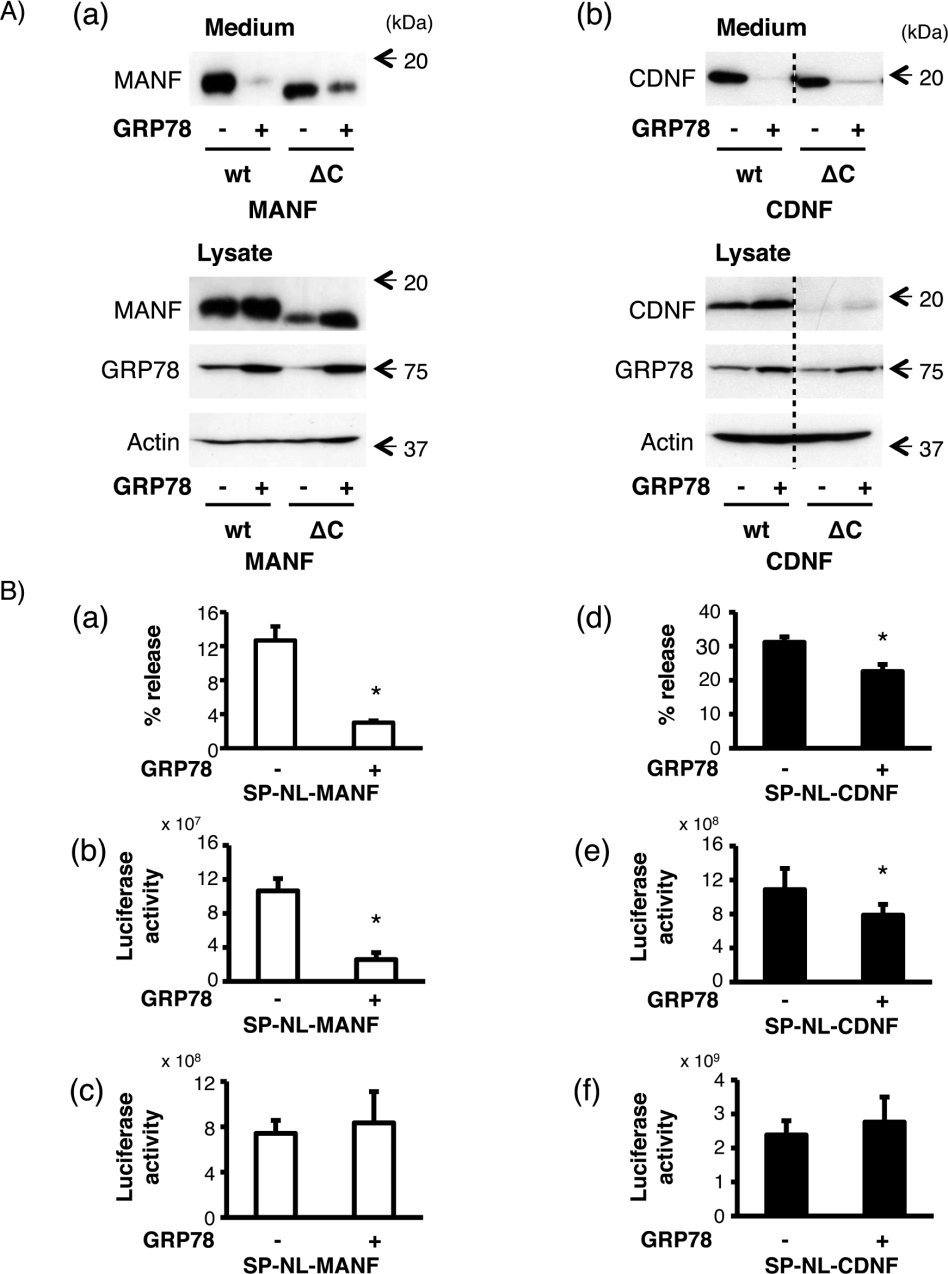
Effects of GRP78 co-expression on the secretion of MANF and CDNF from HEK293 cells. (A) After the transfection of wtMANF or ΔC_MANF_ (A-a) and wtCDNF or ΔC_CDNF_ (A-b) with GRP78 or the empty vector (mock) into HEK293 cells, each indicated protein was detected as described in Fig 2. Representative data of three independent cultures were shown. The broken line represented the boundary line between wtCDNF and ΔC_CDNF_ of the two lanes in the same immunoblotted membrane. (B) After the transfection of SP-NL-MANF (a, b, c) or SP-NL-CDNF (d, e, f) with GRP78 or the empty vector (mock), the luciferase activity of the culture medium (b, e) and cell lysate (c, f) from HEK293 cells expressing SP-NL-MANF or SP-NL-CDNF were measured and relative amounts of secreted SP-NL-MANF and SP-NL-CDNF in each case (a, d) were calculated as described in Fig 2. The values represent the mean ± SEM from six independent cultures. The data were analyzed by Student’s t-test to evaluate the effects of the co-expression of GRP78 on the luciferase activity. The values marked with an asterisk are significantly different from the value of the mock-transfected cells, respectively (p < 0.05).

Comparing the amino acid sequence of MANF and CDNF among several species, the four C-terminal amino acids of MANF (RTDL) and CDNF ([K/Q]TEL) are well conserved. Next, we examined whether the ER-localizing motifs, RTDL and QTEL, affect the secretion of MANF and CDNF, respectively. Accordingly, the secretion levels of wild-type (wt) and its mutant lacking four C-terminal amino acids (ΔC) were investigated with or without wtGRP78 overexpression because the overexpression of GRP78, which is known to be one of the ER resident proteins having this canonical motif (KDEL), attenuated the MANF secretion [20, 29]. As shown in Fig 3A, the amount of MANF in the culture medium was decreased by the overexpression of GRP78, which is consistent with the previous report [29]. In addition to the secretory regulation of MANF in the ER, the secretions of wtCDNF and ΔC_CDNF_ were also decreased by the GRP78-overexpression. From the analysis using the NanoLuc system, the ratio of GRP78-mediated secretory suppression of CDNF was found to be somewhat different from that of MANF (Fig 3B). The secretion of NanoLuc-tagged MANF and CDNF (SP-NL-MANF and SP-NL-CDNF, respectively) from wtGRP78 co-transfected cells were 24.0 ± 1.2% and 72.5 ± 3.2%, respectively, compared with that from mock cells, though this difference was not observed using untagged MANF and CDNF (Fig 3 and Table 2). However, the intracellular amounts of SP-NL-MANF and SP-NL-CDNF showed no statistical significance (110.8 ± 11.4% and 114.6 ± 6.2%, respectively) (Table 2).

**Table 2:** The relative amounts of SP-NL-MANF and SP-NL-CDNF from HEK293 cells co-transfected with mock or GRP78.

	% release		Medium		Lysate	
	mock	GRP78	mock	GRP78	mock	GRP78
**MANF**	100.0 ± 0.9	24.0 ± 1.2	100.0 ± 5.1	24.4 ± 3.5	100.0 ± 5.1	110.8 ± 11.4
**CDNF**	100.0 ± 1.9	72.5 ± 3.2	100.0 ± 5.4	72.9 ± 2.2	100.0 ± 2.3	114.6 ± 6.2

Values show the relative luciferase activities represented in Fig 3B. The relative amounts of secreted SP-NL-MANF and SP-NL-CDNF in each case (Fig 3B a, d) were calculated from the extracellular (Fig 3B b, e) and intracellular (Fig 3B c, f) activities of SP-NL-MANF and SP-NL-CDNF, respectively. Each value represents the mean ± SEM from six independent cultures and was expressed as a percentage of mock cells.

**Fig 4:**
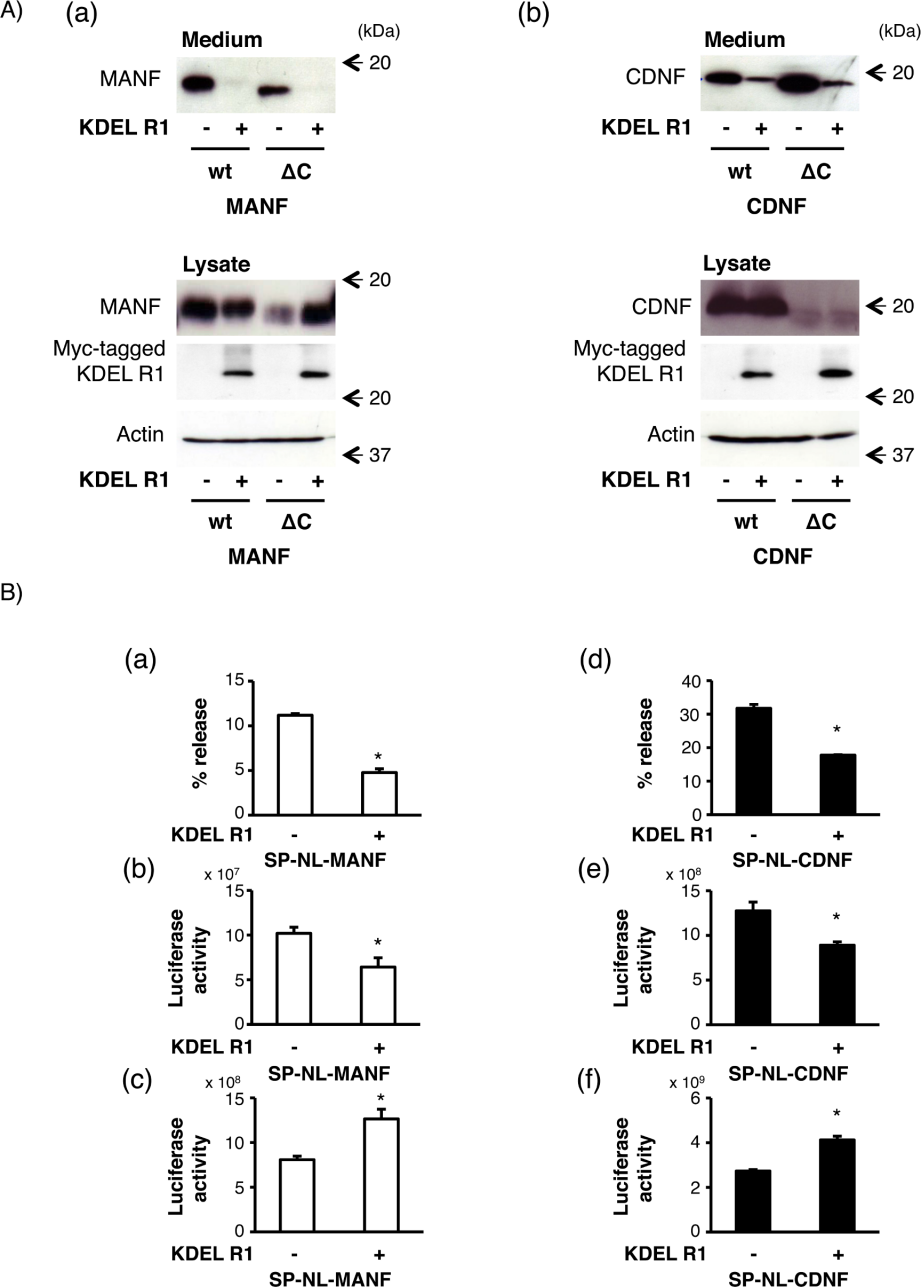
Effects of KDEL receptor1 co-expression on the secretion of MANF and CDNF from HEK293 cells. (A) After the transfection of wtMANF or ΔC_MANF_ (A-a) and wtCDNF or ΔC_CDNF_ (A-b) with KDEL-R1 or the empty vector (mock) into HEK293 cells, the expression of indicated proteins was detected as described Fig 2. Representative data of three independent cultures were shown. (B) Twenty-four hours after the transfection of SP-NL-MANF (a, b, c) or SP-NL-CDNF (d, e, f) with KDEL-R1 or the empty vector (mock), the luciferase activity in each sample was measured and calculated as described in Fig 2. The values represent the mean ± SEM from six independent cultures. The data were analyzed by Student’s t-test to evaluate the effects of the co-expression of KDEL-R1 on the luciferase activity. The values marked with an asterisk are significantly different from the value of the mock-transfected cells, respectively (p < 0.05).

It is known that KDEL-Rs, which exist on *cis-*Golgi network, bind to KDEL-like motif and package ER resident proteins into COPI-coated retrograde transport vesicle [37, 38]. In addition, Henderson *et al*. recently reported that the overexpression of four types of KDEL-Rs decreased the MANF secretion [34]. Therefore, we examined the effects of KDEL-R1 on the MANF and CDNF secretions in our experiment because KDEL-R1 was reported to show the most suppressive effect among them. As shown in Fig 4A, the secretion of wtCDNF was decreased, as was that of wtMANF. Surprisingly, the extracellular levels of ΔC_MANF_ and ΔC_CDNF_ were also decreased to the same extent even though their respective KDEL-like four C-terminal amino acids, RTDL and QTEL, were removed. On the other hand, we observed a significant increase in the intracellular amounts of ΔC_MANF_ but not ΔC_CDNF_ in the current condition (Fig 4A). We further investigated effects of the KDEL-R1 overexpression on the MANF and CDNF secretions using our NanoLuc system. As shown in Fig 4B, the secretions of SP-NL-MANF and SP-NL-CDNF were also reduced by the co-transfection of KDEL-R1 (43.6 ± 1.8% and 59.1 ± 1.4%, respectively, Table 3). On the other hand, the intracellular amounts of SP-NL-MANF and SP-NL-CDNF were significantly increased by KDEL-R1 overexpression (Fig 4B and Table 3). Considering the increase in the intracellular amounts of SP-NL-MANF and SP-NL-CDNF based on the NanoLuc activity, the co-transfection of KDEL-R1 did not hamper their expressions, but was likely to affect the protein stability through the retrograde transport (from the Golgi apparatus to the ER).

**Table 3:** The relative amounts of SP-NL-MANF and SP-NL-CDNF from HEK293 cells co-transfected with mock or KDEL-R1.

	% release		Medium		Lysate	
	mock	KDEL-R1	mock	KDEL-R1	mock	KDEL-R1
**MANF**	100.0 ± 2.3	43.6 ± 1.8	100.0 ± 3.8	50.8 ± 7.4	100.0 ± 2.4	123.5 ± 16.4
**CDNF**	100.0 ± 1.8	59.1 ± 1.4	100.0 ± 4.6	70.0 ± 1.5	100.0 ± 2.1	141.9 ± 5.0

Values show the relative luciferase activities represented in Fig 4B. The relative amounts of secreted SP-NL-MANF or SP-NL-CDNF in each case (Fig 4B a, d) were calculated from the extracellular (Fig 4B b, e) and intracellular (Fig 4B c, f) activities of SP-NL-MANF and SP-NL-CDNF, respectively. Each value represents the mean ± SEM from six independent cultures and was expressed as a percentage of mock cells.

**Fig 5:**
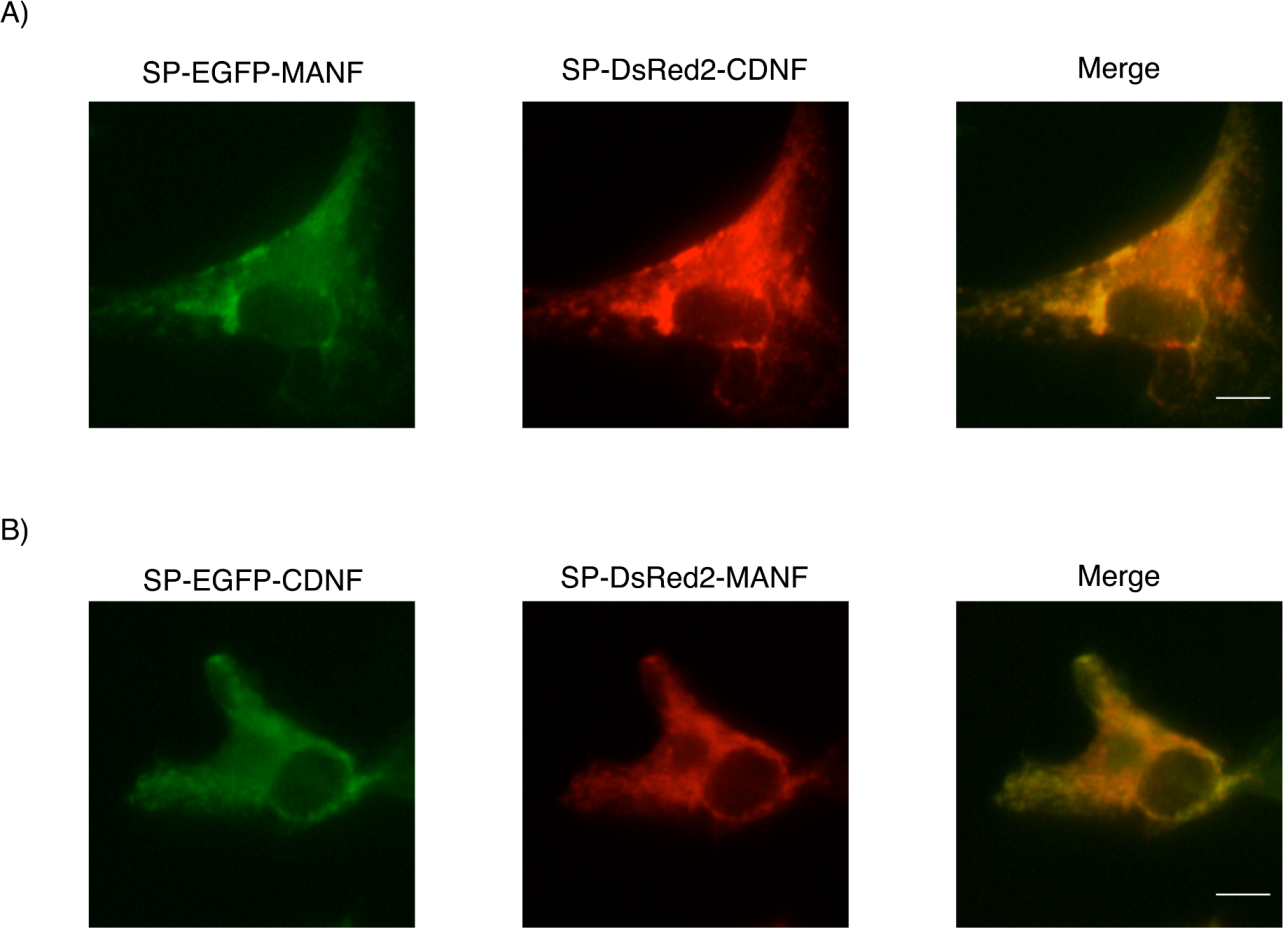
Intercellular localization of MANF and CDNF in COS7 cells. (A, B) Forty-eight hours after transfection of SP-EGFP-MANF and SP-DsRed2-CDNF (A) or SP-DsRed2-MANF and SP-EGFP-CDNF (B) into COS7 cells, the cells were fixed and observed as described in Materials and Methods. Scale bar is 10 μm.

MANF has been reported to be localized in the ER and Golgi apparatus including the peri-nuclear region [10, 28]. As we assumed that the secretory regulation might cause differences between intracellular localization of MANF and CDNF, we investigated the localization of MANF and CDNF in COS7 cells using EGFP- or DsRed2-fusion MANF and CDNF. As shown in Fig 5A and 5B, the intracellular localization of MANF merged well with CDNF.

**Fig 6:**
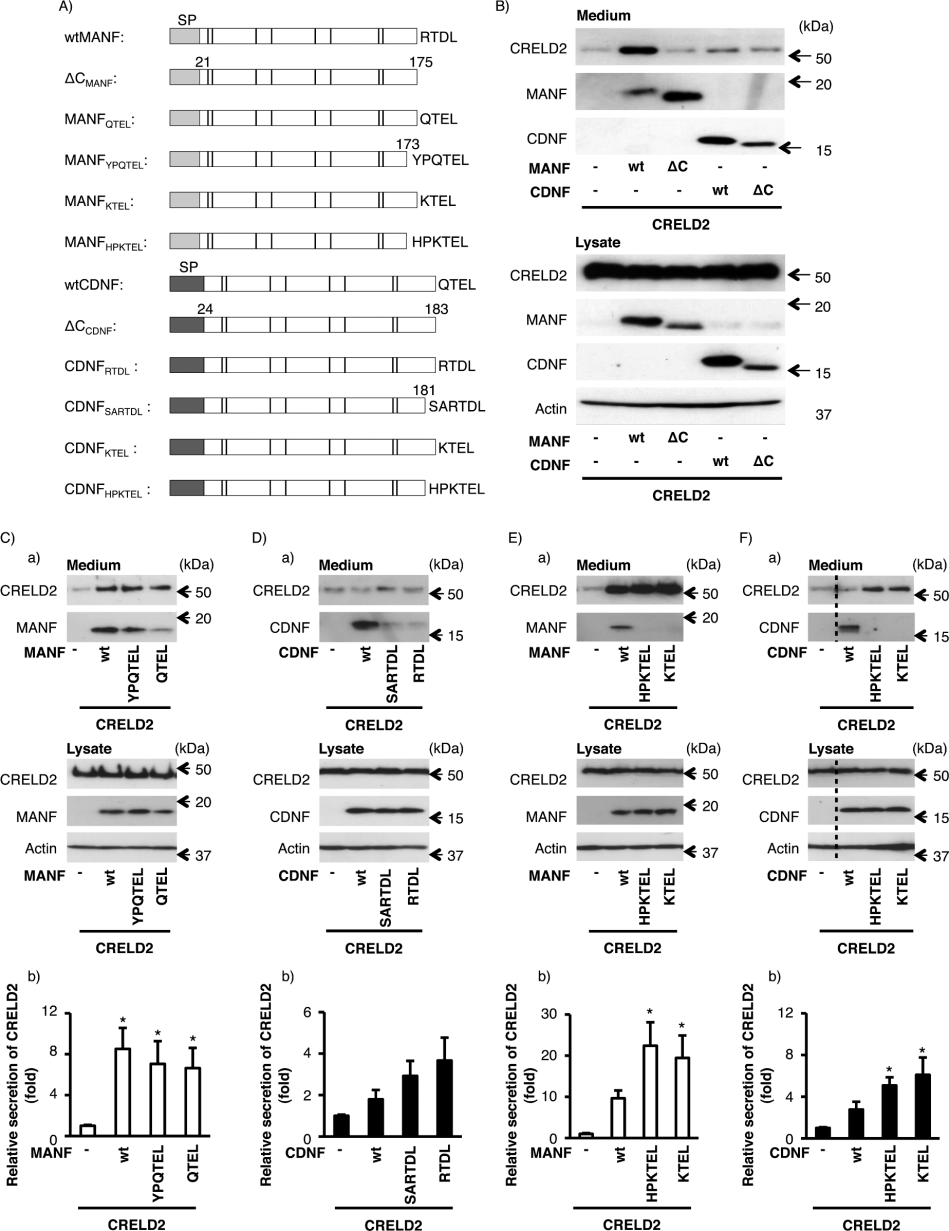
Effects of mouse MANF and CDNF co-expression on the CRELD2 secretion from HEK293 cells. (A) Schematic representation of the mouse MANF and CDNF expression constructs used in this study. SP indicates a signal peptide at the N-terminus of each protein. The cysteines are indicated by bars. The four or six C-terminal amino acids, RTDL, SARTDL, QTEL, YPQTEL, KTEL and HPKTEL, in each construct are shown in capital letter. After co-expression of wtCRELD2 with (B) wtMANF, ΔC_MANF_, wtCDNF or ΔC_CDNF_, (C) wtMANF, MANF_YPQTEL_ or MANF_QTEL_, (D) wtCDNF, CDNF_SARTDL_ or CDNF_RTDL_, (E) wtMANF, MANF_HPKTEL_ or MANF_KTEL_, (F) wtCDNF, CDNF_HPKTEL_ or CDNF_KTEL_, the indicated proteins were detected by western blot analysis as described in Fig 2. Representative data of three independent cultures were shown (B, C-a, D-a, E-a, F-a). The broken line represented the boundary line between the two lanes in the same immunoblotted membrane. (C-F b) Each of bar graphs shows densitometric analyses of the secreted CRELD2 as described in the Materials and Methods. Each value represents the mean ± SEM from 8 (C), 10 (D), 6 (E), 6 (F)-independent cultures. The values marked with an asterisk are significantly different from the values of the mock-transfected cells (p<0.05).

Very recently, we reported that the overexpression of mouse MANF enhanced mouse CRELD2 secretion from HEK293 and COS7 cells [28]. Accordingly, we examined whether mouse CDNF affected the CRELD2 secretion as in the case of mouse MANF. We then co-overexpressed CRELD2 and mouse CDNF with or without the four C-terminal amino acids in HEK293 cells and evaluated the amounts of these proteins inside and outside of the cells, respectively (Fig 6A and 6B). Consistent with our recent report, the overexpression of wtMANF but not ΔC_MANF_ remarkably increased the secretion of wtCRELD2 (Fig 6A and 6B). Surprisingly, mouse wtCDNF and ΔC_CDNF_ hardly influenced the secretion of CRELD2 as well as ΔC_MANF_. To investigate whether the differences in the four or six C-terminal amino acids between mouse MANF and mouse CDNF are responsible for CRELD2 secretion, because Alanen *et al*. suggest that the importance of position-5 and -6 from the C-terminus [39]. Accordingly, we constructed the expression vectors of mouse MANF and CDNF mutants whose C-terminal KDEL-like motifs were exchanged with each other (Fig 6A). As shown in Fig 6A and 6C, we found that mouse MANF having QTEL (MANF_QTEL_) or YPQTEL (MANF_YPQTEL_) increased the CRELD2 secretion to a similar extent. On the other hand, the C-terminal exchanged CDNF (CDNF_RTDL_ and CDNF_SARTDL_) slightly increased the CRELD2 secretion, however it was not statistically significant compared with mock-tranfected cells (p = 0.058) (Fig 6A and 6D). As the four C-terminal amino acids of CDNF in several species including human, chimpanzee and rhesus macaque are “KTEL” but not “QTEL”, we investigated the CRELD2 secretion in the presence of mouse MANF and CDNF having human CDNF-type C-terminal motifs (KTEL and HPKTEL). Interestingly, the co-transfection of mouse MANF_KTEL_ or MANF_HPKTEL_ remarkably increased the CRELD2 secretion, and the increased secretion of CRELD2 by mouse CDNF_KTEL_ or CDNF_HPKTEL_ was almost the same compared with that by mouse wtMANF (Fig 6E and 6F).

## Discussion

The differing features of MANF and CDNF have not been precisely understood, although both of them were reported to show characteristics of cytoprotection for dopamine neurons [17, 23]. As there has been only one report making a direct comparison of characteristics between MANF and CDNF in *Drosophila* [24], we performed a comparative investigation focusing on their secretory mechanisms using western blot analysis and a NanoLuc-based assay. The results obtained in this study are described as follows: (i) CDNF was transported by the COPII-mediated pathway in a similar fashion as MANF [26, 29]; (ii) the secretion of wild-type MANF and CDNF was regulated by ER- or Golgi apparatus-resident proteins, GRP78 and KDEL-R1 in the same manner [20, 26, 29, 34]. However, the GRP78-overexpression attenuated the secretion of SP-NL-CDNF to a much lesser extent (by only 28%) compared with that of SP-NL-MANF. On the other hand, the KDEL-R1 co-expression affected the secretion of SP-NL-MANF or SP-NL-CDNF to the same degree; (iii) mouse CDNF hardly affected the secretion of CRELD2 in contrast to mouse MANF; (iv) the composition of the four C-terminal KDEL-like motifs in MANF and CDNF plays a important role in regulating the CRELD2 secretion; and (v) MANF and CDNF showed similar distribution, suggesting that the difference in ability of CRELD2 secretion was not merely associated with their localization. These findings seem to produce valuable information for clarifying physiological functions of MANF and CDNF.

In this study, we found that the secretion of CDNF was regulated in a similar manner to that of MANF [26, 29, 34]. Many ER-resident proteins have a KDEL-like motif at their C-terminus [32]. KDEL-R recognizes the KDEL-like motif and mediates their trafficking from the Golgi apparatus back to the ER [32, 37, 40]. It is well known that the C-terminal Lys-Asp-Glu-Leu sequence is a canonical KDEL motif that aids in high-affinity binding to the KDEL-R, but a variety of KDEL-like motifs have weaker affinity for this receptor than the KDEL sequence [32]. In parallel with these findings, Glembotski *et al*. first hypothesized that the secretion of MANF was regulated by competition with GRP78 against the KDEL-Rs in the *cis*-Golgi [36], and further demonstrated that MANF retention in the ER was mediated by dual mechanisms, KDEL-R-dependent manner and Ca^2+^-dependent binding to GRP78 [20]. According to this model, MANF is constantly sent back to the ER via a KDEL-R mediated fashion and the retrograded MANF is retained by Ca^2+^-dependent association with GRP78 under conditions of normal Ca^2+^ concentration in the ER. On the contrary, under Ca^2+^-depleted conditions in the ER, the dissociation of the MANF-GRP78 complex is increased followed by the facilitation of MANF secretion. In our experiment using epitope-tagged constructs of SP-Flag-MANF and SP-Flag-CDNF, we showed that the relative amount of secretion of MANF was almost equivalent to that of CDNF. In addition, our present data comparing their secretory regulation by mutant Sar1, KDEL-R1 and wtGRP78 suggest that most of the mechanisms for regulating the secretion of MANF and CDNF could be similar. Like previous studies showing that the overexpression of GRP78 [20, 29] and KDEL-Rs [34] attenuated the secretion of wtMANF, the wtCDNF secretion was also decreased by overexpression of each of the proteins. Similar phenomena were also observed in the cells expressing ΔC_MANF_, ΔC_CDNF_, SP-NL-MANF and SP-NL-CDNF. However, Henderson *et al*. reported that the overexpression of KDEL-Rs reduced the secretion of GFP-tagged MANF but not GFP-tagged MANF lacking the C-terminal RTDL [34]. Although it is unclear why the results were controversial, the GFP-tag at the N-terminus of MANF might cause this discrepancy. The NanoLuc used in this study is a slightly smaller protein than GFP [25]; however, it is likely to influence the secretion of MANF and CDNF to some extent. The secretory profiles of the Flag-epitope (8 aa) tagged MANF and CDNF was almost the same level as those of the wild-types, but SP-NL-CDNF, including NanoLuc, was spontaneously secreted into the medium in greater amounts than SP-NL-MANF. In addition, the attenuated ratio of CDNF secretion affected by GRP78 overexpression was almost abrogated by the insertion of NanoLuc into the CDNF construct. However, our data obtained from the conventional western blot analysis and the sensitive Nanoluc-based assay suggest the secretory regulations of both factors more profoundly, that is these differences obtained from each of the analyses may provide information for the regulatory mechanisms of their secretion. As the KDEL-R1 overexpression attenuated the SP-NL-CDNF secretion more significantly than GRP78 overexpression, each of over-expressed proteins might recognize a different part of the CDNF molecule. On the other hand, the effects of GRP78 overexpression on SP-NL-MANF were comparable to those of KDEL-R1 overexpression. Therefore, the magnitude of the N-terminal structure of each factor to form a complex with GRP78 or KDEL-R1 in the ER and/or Golgi apparatus might be different. In addition, we observed that GRP78 and KDEL-R1 overexpression also down-regulated the secretion of ΔC_MANF_ and ΔC_CDNF_ lacking the C-terminal KDEL-like motif. These results suggest that ER chaperones, including GRP78, with the canonical KDEL-motif responsible for ER retention might form a complex with MANF and CDNF. On the other hand, it is unlikely that KDEL-R1 directly recognizes their four C-terminal amino acids, RTDL and [Q/K]TEL though they are well conserved among several species. It is considered that the well-conserved KDEL-like motifs in MANF and CDNF have some functions; however, the recognition of proteins having KDEL-like motifs by KDEL-Rs might be more complicated. Henderson *et al*. demonstrated that the effects of four types of KDEL-Rs on the MANF secretion varied [34]. Therefore, it is necessary to characterize not only the C-terminal KDEL-like motifs but also other domains of MANF and CDNF in more detail. Our result concerning the intracellular distribution of MANF and CDNF using EGFP and DsRed2 showed that both factors co-localized in the ER and Golgi apparatus as well as in the peri-nuclear region of COS7 cells. Meanwhile, our current study also implies that KDEL-R1 is unlikely to recognize the C-terminal KDEL-like motifs of MANF and CDNF. Therefore, we consider that MANF and CDNF may form several types of transported complexes during bidirectional ER-Golgi transports, although it is unclear whether MANF and CDNF form a complex with other proteins in the ER and/or Golgi apparatus are transported by the same cargos.

We performed further study of the molecular features of mouse MANF and CDNF. We have been investigating the function of CRELD2, which was previously identified as a new ER stress-inducible protein under pathophysiological conditions, and very recently reported that mouse wtMANF but not ΔC_MANF_ increased the secretion of CRELD2 [27, 28, 41]. As wtCDNF and ΔC_CDNF_ hardly affected the CRELD2 secretion (Fig 6), we considered whether the C-terminal KDEL-like motif in MANF is responsible for the CRELD2 secretion. Therefore, the KDEL-like motifs of MANF and CDNF were exchanged with each other to make MANF_QTEL_ and CDNF_RTDL_. As a result, MANF_QTEL_ increased the CRELD2 secretion by co-expression as the same level as wtMANF, whereas CDNF_RTDL_ promoted its secretion to a lesser extent. As Alanen *et al*. have reported that the six C-terminal amino acids play an important role in determining the ER localization and recognizing the KDEL-like motifs [39], we tested the effects of the six C-terminal amino acids of mouse MANF and CDNF on the CRELD2 secretion. However, the additional 2-amino-acid replacement marginally influenced the CRELD2 secretion furthermore. On the other hand, effects of the exchange of human CDNF-type C-terminal motifs (KTEL and HPKTEL) for those of mouse MANF were more remarkable. Co-transfection of MANF_KTEL_ or MANF_HPKTEL_ almost doubled the CRELD2 secretion compared with mouse wtMANF, and the increased levels of CRELD2 secretion by CDNF_KTEL_ or CDNF_HPKTEL_ was comparable with those by mouse wtMANF. In our previous report, we demonstrated that the mouse MANF having a canonical four C-terminal amino acids (MANF_KDEL_) markedly elevated the CRELD2 secretion under the same experimental condition [28]. Collectively, the positive charge such as lysine at the position-4 from the C-terminus could be responsible for regulating the retention and secretion of ER resident proteins. To support this idea, we observed that the amount of secreted CRELD2 promoted by human CDNF was comparable with that by mouse wtMANF (S1 Fig). As the four C-terminal amino acids of CDNF (KTEL) is conserved among several species including human, chimpanzee and rhesus macaque, this finding seems to be valuable information to uncover the molecular features of CDNF. We therefore consider that characterization of these differences among several species in addition to analysis for other domains of MANF, CDNF and CRELD2 proteins might give new insights into understanding cytoprotective abilities of MANF and CDNF.

MANF, CDNF and CRELD2 are suggested to contain a PDI-like motif, CXXC, in their C terminal regions, such as ^127^CKGC^130^ in MANF, ^132^CRAC^135^ in CDNF and ^259^CVGC^262^ in CRELD2. These proteins are therefore considered to participate in the quality control of proteins in the ER. Indeed, Hartley *et*. *al* reported that MANF and CRELD2 had substrate specificity to form a complex with misfolded proteins, but MANF did not possessed the PDI-like activity in contrast to CRELD2 [42]. However, it might be caused by rapid degradation of the mutated MANF-bait complex during cell homogenation. Further study is required to determine whether MANF has PDI-like activity.

In our present study, we showed that mouse CDNF shared secretory regulation with mouse MANF; however, the mouse CDNF overexpression hardly affected the co-transfected CRELD2 secretion, whereas mouse MANF and human CDNF did. Accordingly, we presume that relationships of MANF, CDNF and CRELD2 might differ among several species, and it is intriguing whether changes in each of the expressions and subcellular localizations might co-operatively influence the ER homeostasis (e.g., a quality control of certain secretory and transmembrane proteins) under ER stress conditions. Therefore, clarifying roles of MANF, CDNF and CRELD2 under some pathophysiological conditions may give us new insight into the progression of ER stress-related diseases and a new strategy for finding cures for these diseases.

## Supporting Information

S1 Fig Effect of human CDNF co-expression on the CRELD2 secretion from HEK293 cells. A) Twenty-four hours after the transfection of CRELD2 with human wild-type CDNF (wt hCDNF) or the empty vector (mock) into HEK293 cells, the culture medium was replaced with fresh serum-free medium and the cells were cultured for an additional 12 h. The amounts of the indicated proteins in the cell lysate and culture medium were detected by western blot analysis as described in the Materials and Methods. Representative data of three independent cultures were shown. The human wild-type CDNF (wt hCDNF) gene was cloned from cDNA derived from HEK293 cells and inserted into the pcDNA3.1 vector. B) Each of bar graphs shows densitometric analyses of the secreted CRELD2 as described in the Materials and Methods. Each value represents the mean ± SEM from six independent cultures. The values marked with an asterisk are significantly different from the values of the mock-transfected cells (p<0.05).
(TIF)

## Abbreviations

Armet: arginine-rich, mutated in early stage of tumors
CDNF: cerebral dopamine neurotrophic factor
CRELD2: cysteine-rich with EGF-like domains 2
EGFP: enhanced green fluorescent protein
ER: endoplasmic reticulum
ERAD: endoplasmic reticulum associated degradation
GRP78: glucose-regulated protein 78 kDa
MANF: mesencephalic astrocyte-derived neurotrophic factor
