# Supplementary figures and images for: A Comparative Analysis of the Molecular Features of MANF and CDNF
Source: PLoS One. 2016 Jan 28;11(1):e0146923. doi: 10.1371/journal.pone.0146923 (PMC4731063; doi:10.1371/journal.pone.0146923)

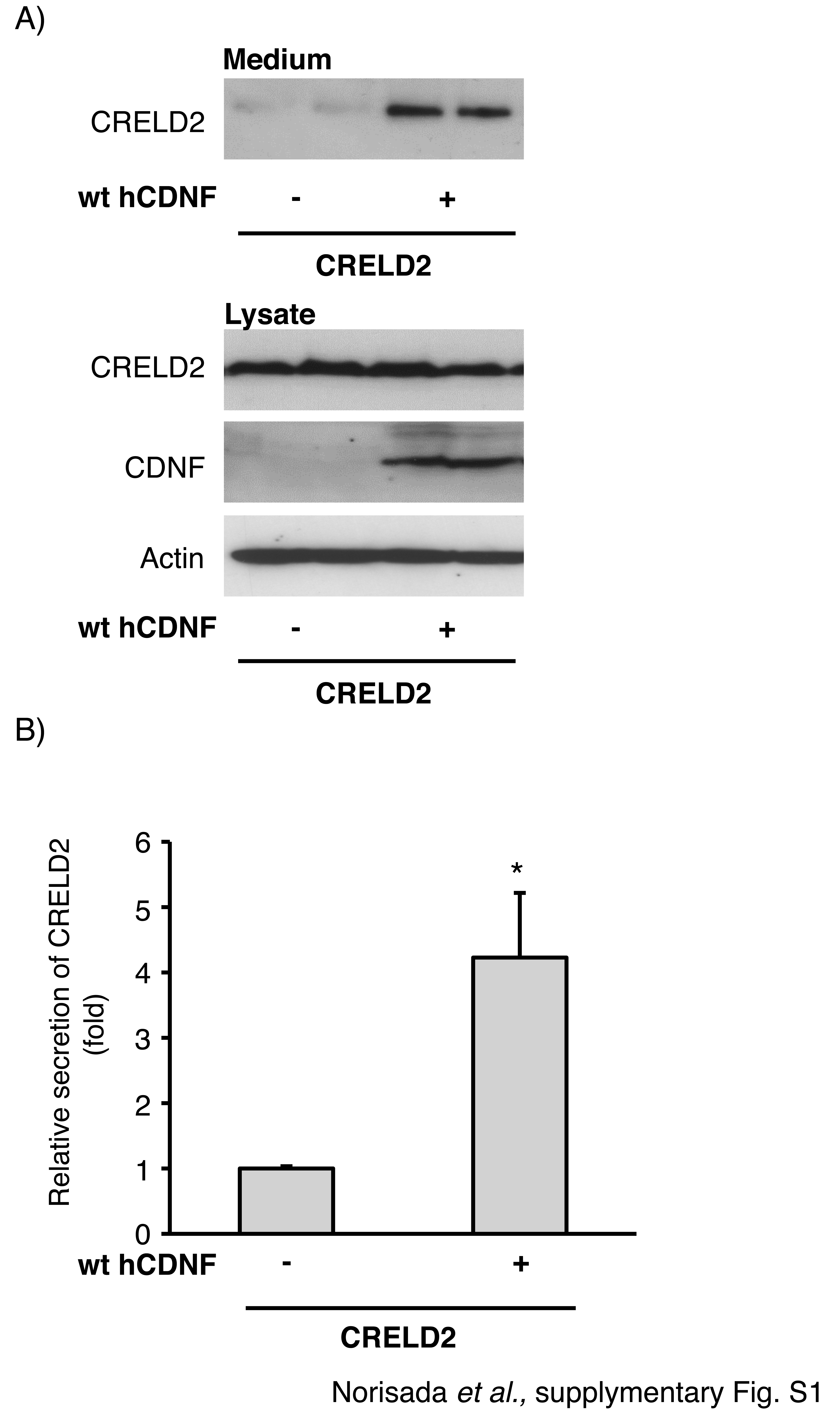

Supplement: S1 Fig — A) Twenty-four hours after the transfection of CRELD2 with human wild-type CDNF (wt hCDNF) or the empty vector (mock) into HEK293 cells, the culture medium was replaced with fresh serum-free medium and the cells were cultured for an additional 12 h. The amounts of the indicated proteins in the cell lysate and culture medium were detected by western blot analysis as described in the Materials and Methods. Representative data of three independent cultures were shown. The human wild-type CDNF (wt hCDNF) gene was cloned from cDNA derived from HEK293 cells and inserted into the pcDNA3.1 vector. B) Each of bar graphs shows densitometric analyses of the secreted CRELD2 as described in the Materials and Methods. Each value represents the mean ± SEM from six independent cultures. The values marked with an asterisk are significantly different from the values of the mock-transfected cells (p<0.05). (TIF) [file pone.0146923.s001.tif]
